# Eye Plaque Brachytherapy for Choroidal Malignant Melanoma: A Case Report on the Use of Innovative Technology to Expand Access, Improve Practice, and Enhance Outcomes

**DOI:** 10.7759/cureus.54572

**Published:** 2024-02-20

**Authors:** Paul Mobit, Claus Chunli Yang, Mary R Nittala, Rui He, Hiba Z Ahmed, Gary Shultz, Albert Lin, Srinivasan Vijayakumar

**Affiliations:** 1 Radiation Oncology, University of Mississippi Medical Center, Jackson, USA; 2 Radiation Oncology, G.V. (Sonny) Montgomery VA Medical Center, Jackson, USA; 3 Ophthalmology, University of Mississippi Medical Center, Jackson, USA; 4 Radiation Oncology, Cancer Care Advisors and Consultants LLC, Jackson, USA

**Keywords:** dose-volume histogram, overall survival, eye plaque interstitial brachytherapy, proton beam radiotherapy, choroidal malignant melanoma

## Abstract

Our institute established an eye plaque interstitial brachytherapy (EPIBT) program in 2007 using the Collaborative Ocular Melanoma Study (COMS) eye plaque. In this case report, we demonstrated an eye plaque treatment planned and executed using Eye Physics Plaque (Los Alamitos, CA) for a 72-year-old male patient with an extra-large tumor with a maximum width of 18.6 mm and height of 13.7 mm. The use of a customized eye plaque, manufactured through three-dimensional (3D) printing, has empowered us to plan and administer treatment for this patient with uveal melanoma. Without this option, enucleation, an option declined by the patient, or proton beam therapy (PBT), which the patient was unwilling to pursue in another state, would have been the alternative course of action. We were able to use more than one activity of the I-125 seeds, which enabled us to shape and reduce the dose to normal surrounding structures at risk within the orbit and in the vicinity of the orbital cavity. Using the dose evaluation tools available with the modern treatment planning system, we reduced the prescription dose from 85 to 70 Gy, with D90 of 140 Gy, thereby providing effective treatment and limiting risk organ doses. In summary, we were able to dose-deescalate without compromising the chances of controlling retinal/scleral tumors. The patient is doing well from a recent follow-up visit 12 months after the eye plaque brachytherapy treatment. The tumor was 4.80 mm high, 1/3 of the original height, and vision is back to 20/60, demonstrating a successful treatment.

## Introduction

The most common intraocular cancer is uveal melanoma, with about 2,500 new cases per year in the United States, followed by retinoblastoma, with about 350 patients yearly. The treatment of uveal melanoma initially was enucleation, but the Collaborative Ocular Melanoma Study (COMS) randomized trial comparing brachytherapy with enucleation for choroidal melanoma showed no survival differences between the two treatment options [1,2]. The University of Mississippi Medical Center has established an eye plaque interstitial brachytherapy (EPIBT) program since 2007, and our catchment area includes the whole state of Mississippi. Our program treats 6-12 patients yearly using iodine-125 radioactive seeds. It was also shown through the COMS study that for small to medium-sized ocular melanomas, EPIBT has the same local control or better outcomes compared to enucleation. These are for tumors with a maximum apical height of 5 mm or less and basal diameter of 16 mm or less.

Proton beam therapy (PBT) is also a well-established treatment option for ocular melanoma, and many studies have been undertaken to compare PBT and EPIBT [3]. These techniques (PBT and EPIBT) have similar efficacy. Still, there are differences between adverse outcomes associated with them [4], depending on the tumor’s location and size relative to the fovea, lens, and optic nerve. For example, a systematic comparison between the dose distributions delivered in PBT and EPIBT techniques was undertaken by Trofimov et al. [5]. The results indicate that for lesions located superiorly, with a combined sum of apical height and basal diameter measuring 21 mm, EPIBT was superior to PBT. In contrast, PBT is preferable for larger tumor sizes and heights exceeding 10 mm.

Brachytherapy is one of the oldest forms of radiotherapy (RT), and it involves the placement of radioactive sources near or inside the tumor to be treated. The radioactive sources are either permanently implanted (as is the case in early-stage, favorable risk prostate cancer [PC] using radioactive iodine-125 seed implant) or temporally, as in the case of high-dose-rate (HDR) brachytherapy used for the treatment of gynecological cancers. EPIBT is, therefore, a temporary brachytherapy technique where the radioactive sources are placed in specially designed plaques that are then sutured in position behind the ocular lesion to provide a therapeutic radiation dose for a specified time.

The treatment period ranges from three to seven days, depending on the size of the plaque and the dose prescribed [6]. The patient usually goes home after the implantation and then returns for the removal of the plaque three to seven days later, depending on the clinical treatment plan. The patient and the live-in family members are educated on the radiation safety measures and the principles of *as low as reasonably achievable* (ALARA). ALARA principle means that radiation exposure to any person should be eliminated or minimized even when the dose is small, considering economic and social factors. When we cannot eliminate radiation exposure, then three basic protective measures in radiation safety, time, distance, and shielding, should be used to reduce radiation exposure.

All patients with eye plaques and their families receive education on implementing radiation safety precautions. Patients are required to sleep in a separate room from any family member and must avoid being near children or pregnant family members. The patient is to remain isolated in their room to minimize radiation exposure to family members. If they need to be near a family member for any reason, the patient must use the lead eye shield, which effectively reduces exposure by 90%. Radiation exposure diminishes with distance from the source according to the inverse square law. Therefore, staying on the opposite side of the patient and maintaining a distance of at least 3 feet or more helps minimize exposure. The total radiation exposure is directly proportional to the time spent with the patient, emphasizing that reducing the time spent near the patient effectively lowers radiation exposure.

## Case presentation

**Figure 1:**
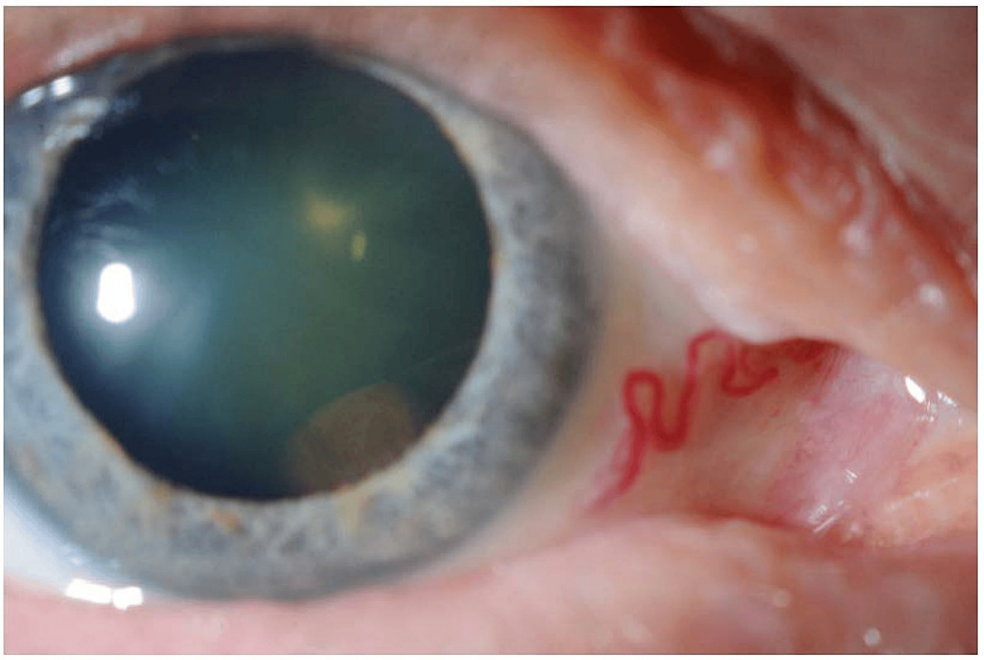
A slit lamp photo showing an inferonasal pigmented choroidal elevated lesion just posterior to the lens with a feeder vessel.

A 72-year-old male with a four-month history of blurry vision in the right eye (20/40 best-corrected visual acuity) presented to the referring physician with a superotemporal peripheral visual field defect. A slit lamp examination revealed an inferonasal retrolental pigmented mass extending from 2 to 6:30 o'clock, touching the lens, as depicted in Figure 1.

**Figure 2:**
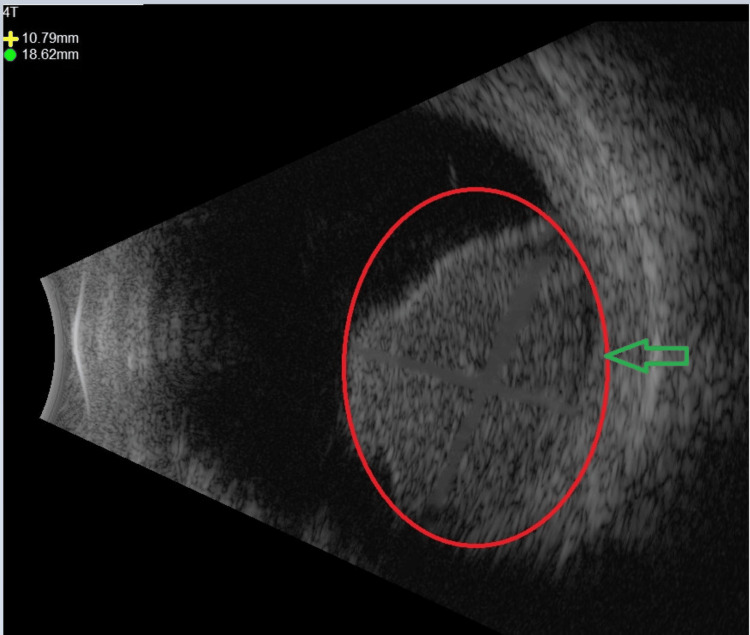
B-scan ultrasonography, at 4 o’clock transverse orientation, showing a classic collar-button appearance.

**Figure 3:**
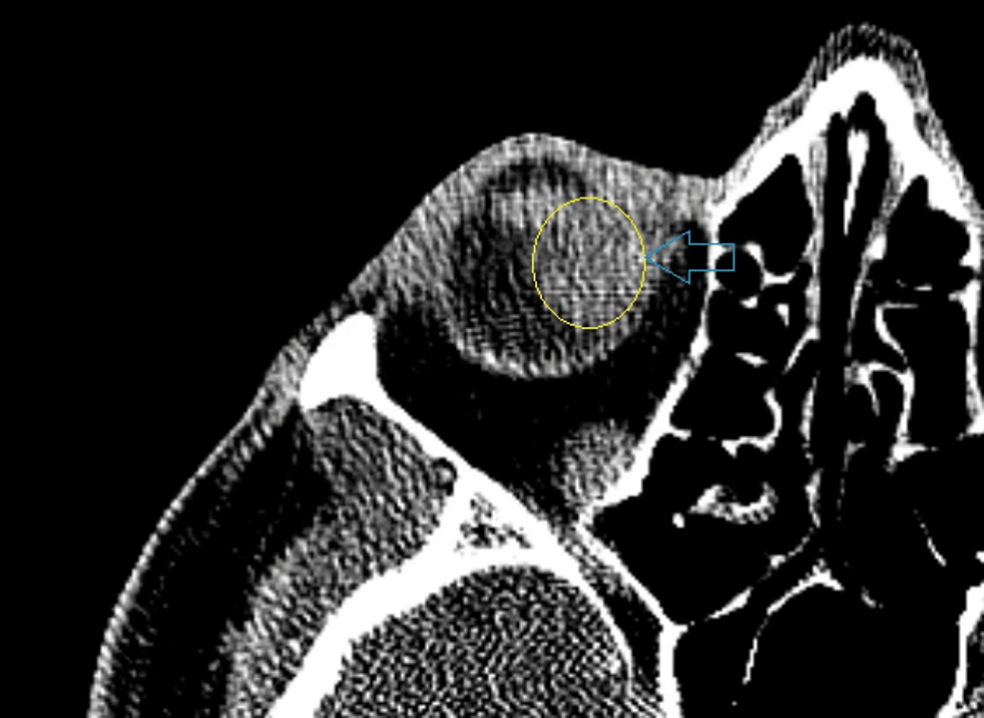
Computed tomography orbit, axial cut of uveal melanoma in the right (R) eye.

The mass was large enough to obscure the view of the inferonasal retina on indirect ophthalmoscopy. On B-scan ultrasonography, the lesion measured 13.7 mm in height, 17.8 mm longitudinally, and 18.6 mm transversely (Figure 2). One mm cut CT scan with contrast showed a 1.4 cm mildly enhancing soft tissue mass within the medial posterior aspect of the right globe, consistent with the slit examination (Figure 3).

The patient desired to preserve his vision and declined enucleation. Proton beam RT was offered elsewhere out of state; however, he was not interested in seeking medical care out of state. Consequently, the patient elected to undergo brachytherapy for lesion destruction.

The dilemma

This particular patient presented three significant challenges.

For this patient, the maximum dimension of the base of the tumor was 19 mm. COMS plaque ranges in size from 10 to 22 mm. Per the COMS protocol, the plaque should have a 2-3 mm margin around the lesion. Therefore, adding 4-6 mm to the base dimension will give rise to a plaque size of at least 23 mm but preferably 25 mm. Therefore, we could not treat this patient using the COMS plaque as 22 mm is the maximum size of the COMS plaque available. The second significant problem with the patient was that the lesion's height was 13.7 mm, which was 3.7 mm more than the standard practice under the COMS protocol. Third, the patient did not want to travel out of state for PBT and was not interested in enucleation.

Based on the National Comprehensive Cancer Network (NCCN) guidelines, Version 1 [7], the treatment options for his tumor should be RT with protons or other charged particle beams, which are not available in our state. This left us with stereotactic radiosurgery (SRS) or enucleation as the alternatives, with the patient expressing a preference against the latter. SRS is an RT procedure that uses accurate tumor targeting to deliver large doses in a single fraction to destroy cancer. SRS is only possible in select tertiary centers and is not available here at the University of Mississippi Medical Center, and this patient did not wish to travel out of state for treatment for this option. Given this, we were obliged to look for alternative solutions to treat this patient’s ocular melanoma.

**Figure 4:**
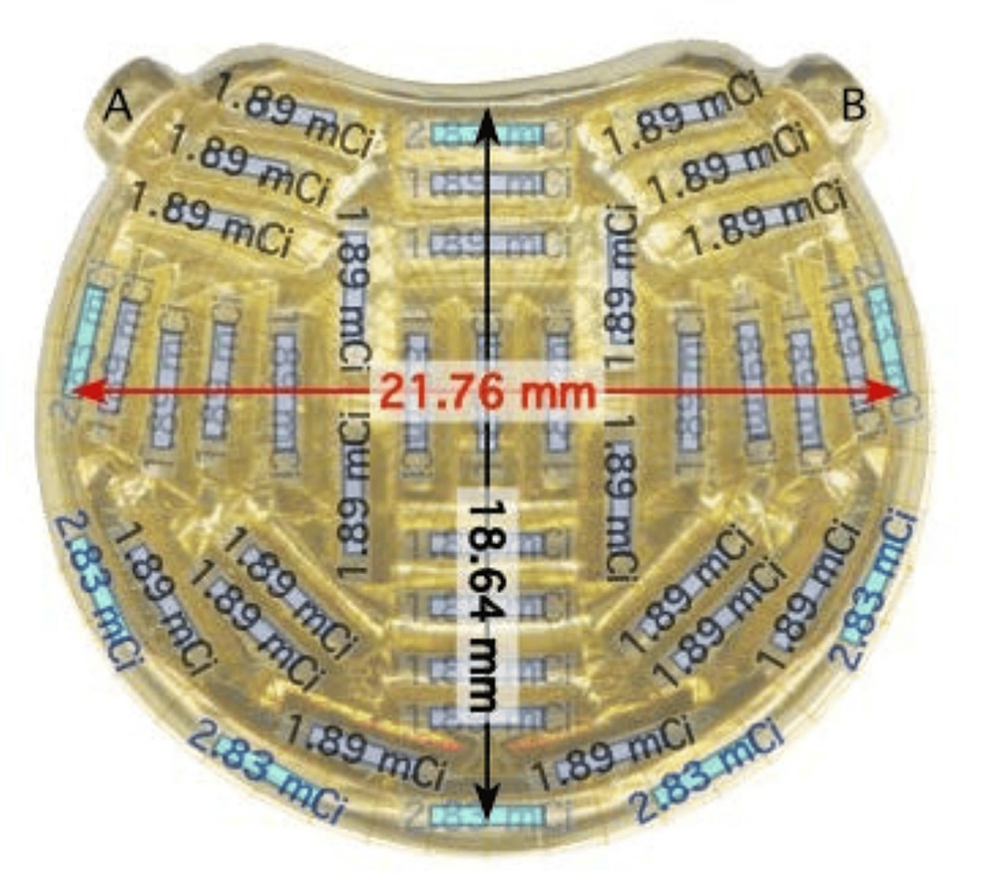
(A) and (B) The three-dimensional (3D) printed eye plaque model with suture anchor positions. This plaque is designed by Eye Physics LLC ( Los Alamitos, CA). mCi, millicurie

We contacted Eye Physics, LLC (Los Alamitos, CA) to design an eye plaque and generate a brachytherapy plan for this patient. The advantage of *Eye Physics plaques* is that they are customized for each patient based on three-dimensional (3D) printing using 18k gold (Figure 4). The plaques can cover a much larger tumor base than the original COMS plaque. The thickness of these plaques ranges from 1.5 to 2.0 mm as opposed to the COMS plaques, which are 5 mm thick.

The radioactive sources are individually collimated in the plaque so that there is a significant reduction in dose to some critical structures such as the sclera.

The RT was planned using the eye physics plaque simulator, a 3D treatment planning software that accepts computed tomography (CT) or magnetic resonance imaging (MRI) scans as input. Critical structures such as the tumor, optic nerve, lens, and sclera can be contoured as in standard external beam RT planning systems, and critical structures’ doses can be determined as in a standard RT planning exercise to which radiation oncologists and medical physicists are familiar due to routine use. The plaque simulation software version 6.9.5 allows the planner to incorporate ultrasound images and slit photography to be fused and integrated with the CT or MRI images. In addition, the software can model radioactive sources/seeds of I-125, Pd-103, Ir-192, and Ru-106 used in plaque therapy for ocular tumors. With the use of CT and MRI, some of the advanced RT planning evaluation tools, such as dose volume histogram (DVH) and dose surface histogram (DSH), can now be used to evaluate the dose distribution to the tumor as well as to the standard structures at risk such as the ipsilateral and contralateral retina, ciliary structures, cornea, lens, and optic nerve. Another advantage of using the eye physics plaque simulator is that more than one radioactive source's activity for the implant can be used, which gives more degrees of freedom in the optimization. 

Per COMS protocol, the prescription dose for the eye plaque is always 8,500 cGy, and the prescription point is 5 mm, even if the tumor apical height is less than 5 mm. However, for tumors with an apical height of more than 5 mm, the actual maximum height or thickness is used as the prescription point or depth. For this patient's treatment, the height was 13.7 mm. Therefore, in the plan's first iteration, the prescription point was set to 13 mm, and the dose was 8,500 cGy. 

**Figure 5:**
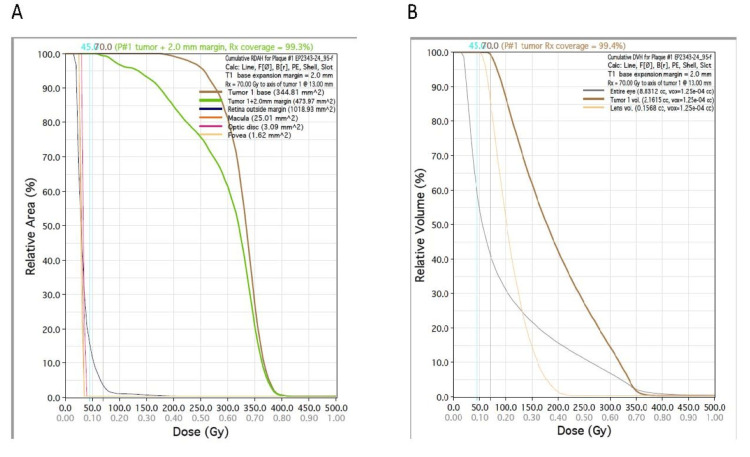
Dose-surface histogram: (A) plaque simulator retina dose-area histogram and (B) plaque simulator DVH. Gy, gray; mm, millimeter; cc, cubic centimeter; RDAH, retina dose-area histogram; DVH, dose-volume histogram; Rx, prescription; vox, voxel based

Figure 5 shows the DSH for the retina on the left and the DVH for the entire eye, lens, and tumor on the right side of the figure.

**Figure 6:**
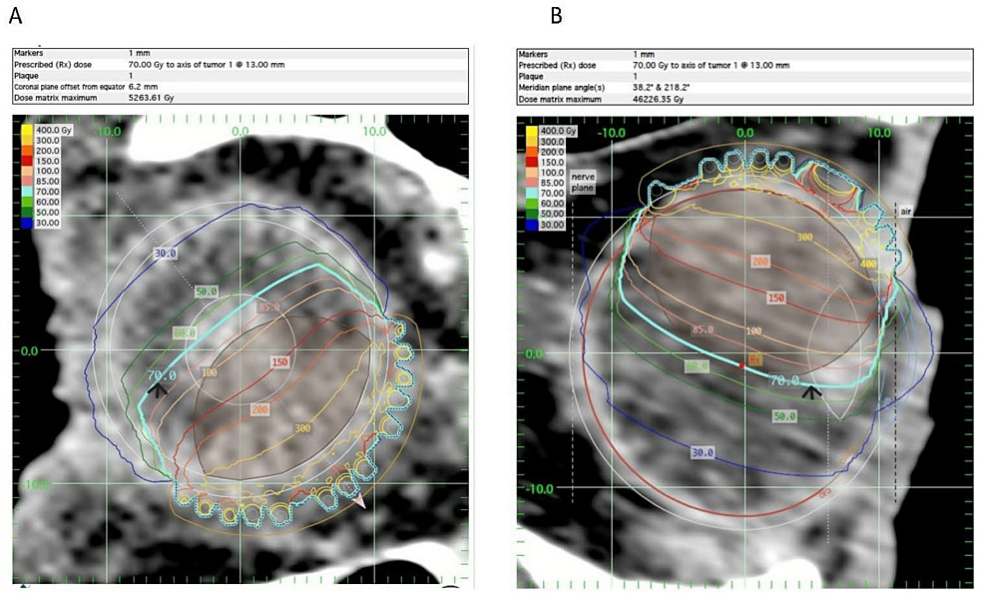
Dose distribution through the coronal and meridian axes: (A) plaque simulator isodose plot for the coronal plane and (B) plaque simulator isodose plot for the meridian plane. Gy, gray; Rx, prescription

Figure 6 shows the dose distribution through the coronal and meridian axes. 

The generated plan was acceptable for treatment, except that the maximum retina dose was 500 Gy and the maximum sclera dose was 480 Gy. These are extremely high-risk organ doses, and some practitioners have set a maximum scleral or retina dose of 400 Gy to proceed with the eye plaque treatment. We had an intradepartmental and interdepartmental peer review board to find a suitable solution. The options were to either decrease the dose to ensure the maximum sclera dose is reduced to 400 Gy or to conduct a staged insertion with a break of two to four weeks before the second insertion. If one were to do a staged implantation, determining the required initial and second prescription doses becomes highly uncertain. To figure out the fraction doses and time interval between the two fractions for staged RT from the radiobiology point of view, preliminary calculations and data fitting were performed based on the incomplete-repair model [8]. 

Our result shows that if we split a single fraction of 85 Gy into two fractions and wait for a certain amount of time (interval time), the fraction dose and interval time we needed to get the same biological equivalent dose (BED) as a single fraction of 85 Gy can be from 45 to 50 Gy, with an interval time of 22 to 76 days. The maximum sclera dose can be from 300 to 400 Gy for a fraction dose of 50 and 250 Gy to 350 Gy for a 45-Gy fraction dose.

As an alternative, the team decided to analyze the dose heterogeneity within the tumor volume. We observed that one-third of the tumor in height received 300 Gy, half received 200 Gy, and three-fourths received 150 Gy. We also observed that D90 for the tumor plus a 2 mm margin (planning target volume [PTV]) was 225 Gy, and D95 was 170 Gy, which is twice the prescription dose. If we reduced the prescription dose to 70 Gy, the dose received by 90% of the volume (D90) would be about 191 Gy, and D95 would be about 140 Gy, which is still a substantial dose. After another tumor board review, the team settled on reducing the prescription dose from 85 to 70 Gy in single-fraction RT, leading to a maximum scleral and retinal dose of slightly less than 400 Gy. It is also important to note that according to the American Brachytherapy Society and the Ophthalmic Oncology Task Force guidelines [9], dose prescriptions for uveal melanoma typically range from 70 to 100 Gy to the apex of tumors. So, we were still within the *standard* criteria with this prescription dose.

Radiation safety

The United States Nuclear Regulatory Commission (NRC) permits the release of a patient with radioactive activity if the estimated effective dose to other individuals (general public) is less than 5 mSv (500 mrem) or if the implanted radioactive source is less than 2 mCi for I-125. In a standardized implant for retinal melanoma, the implanted activity to treat the lesion is recommended to be 85 Gy, and the total activity required to treat the tumor of 13 mm in height is 108 mCi, about 50 times higher than the patient’s release criteria based on implanted activity. Based on an occupancy factor of 0.25, the estimated exposure for someone who would stay close was about 4.8 mSv. This was very close to the recommended guidelines, and we also considered whether to treat the patient as an inpatient or release the patient home after recovery from anesthesia. When we reduced the prescription dose to 70 Gy, the calculated exposure for 168 hours was 4 mSv, which was still relatively high. We opted to purchase a lead eye shield. We released the patient to return home, providing thorough education and instructions to adhere to radiation safety precautions. This includes staying in a separate room within his home for the duration of the implant and using the lead eye shield for the few hours he might spend with his family.

## Discussion

Outcome studies for 1,224 patients who underwent either PBT or EPIBT for uveal melanoma showed that PBT was associated with inferior survival outcomes compared to EPIBT [4]. The two-year overall survival for EPIBT-treated patients was 97% compared to 93% for PBT. The five-year overall survival was 77% for EPIBT versus 51% for PBT [4]. For the case reported here, B-scan ultrasonography six months after treatment was performed and showed a tumor height of 7.8 mm, demonstrating about 43% reduction in height, in line with expected tumor shrinkage of 50% within six to nine months of treatment and no basal expansion. Based on his current condition, no further brachytherapy treatments are planned. The patient is doing well, as evidenced by a recent follow-up visit conducted 12 months after the eye plaque brachytherapy treatment. The tumor now measures 4.80 mm in height, representing one-third of the original height, and vision has improved to 20/60, demonstrating a successful treatment one year after the implant.

## Conclusions

The capability to plan the eye plaque treatment using a customized eye plaque manufactured through a 3D printing technique has allowed us to plan and treat a patient with a very large uveal melanoma lesion. Without this option, the alternative might have involved enucleation or PBT. In addition, using two or more activities has enabled us to shape and reduce the dose to surrounding structures at risk within the orbit and in the vicinity of the orbital cavity. Using the dose evaluation tools available with modern treatment planning, we generated DVH and DSH to determine the holistic dose coverage of the tumor and PTV as well as the sensitive surrounding normal tissues. 

Without these tools, we would not have been able to calculate the maximum critical structure dose. This would have led to a decision not to offer the radioactive seed implant brachytherapy to this patient. With this experience and the ability to generate DVH/DSH information, we can dose-deescalate without compromising the chances of controlling retinal/scleral tumors in the future.
